# The neural basis of intergroup threat effect on social attention

**DOI:** 10.1038/srep41062

**Published:** 2017-01-25

**Authors:** Yujie Chen, Yufang Zhao, Hongwen Song, Lili Guan, Xin Wu

**Affiliations:** 1 School of Psychology, Southwest University, Chongqing, People’s Republic of China; 2 Key Laboratory of Cognition and Personality, Ministry of Education, Chongqing, People’s Republic of China; 3 School of Humanities and Social Science, University of Science and Technology of China, Anhui, People’s Republic of China; 4 School of Psychology, Northeast Normal University, Changchun, People’s Republic of China

## Abstract

Previous gaze-cuing studies found that intergroup threat is one of the modulators of gaze cuing. These findings indicate that intergroup threat would gate social attention by activating a network resembling that is thought to be involved in drawing or/and holding attention. The present study tested this hypothesis using a gaze-cuing task in which a particular in-group participants observed threatening out-group and nonthreatening out-group gazes, while undergoing functional magnetic resonance imaging. As expected, greater gaze cuing effect only emerged for threatening out-group when the in-group participants only felt inergroup threat from that out-group. Behaviorally, we found intergroup threatening out-group gazes did not draw attention faster than nonthreatening in-group gazes does. However, participants took more time to suppress the influence of the gaze direction of threatening out-group gazes, compared to nonthreatening in-group gazes, in the incongruent condition, which means intergroup threatening gaze holds attention longer than nonthreatening gaze does. Imaging results demonstrated that threatening cues recruited a fronto-parietal network, previously implicated in holding attention and execution functions. Our results, therefore, suggest that the mechanisms underpinning gaze cuing evolved to be sensitive to intergroup threatening stimuli, possibly because it is hard to disengage from such intergroup threatening cues once they are detected.

The tendency of human beings to shift their attention to the location of another person’s direction of gaze seems to be related to a well-known ability called gaze cuing of attention^1^,^2^. This prioritized processing of gaze stimuli can help us understand the future intentions and attitudes of other individuals, so that we can change our behavior to respond to beneficial or dangerous stimuli^3^,^4^.

There is strong evidence that human observers tend to respond faster to gaze-congruent targets than to gaze-incongruent targets, a phenomenon which is known as the gaze-cuing effect^5^,^6^,^7^,^8^. Importantly, the emergence of this gaze-cuing effect in human observers is sensitive to social variables related to both the person being observed, the observer, and the relationship between them^9^,^10^,^11^,^12^. Although the few studies investigated the role of group membership on gaze cuing focused on covert orienting, a recent study provided evidence that also the overt orienting is modulated by this variable^13^. Specially, a common modulator of the gaze-cuing effect is specifically related to intergroup threat. For instance, when individuals observed the gaze cues of both high-status individuals and low-status individuals in studies, a greater gaze-cuing effect was found only for the high-status individuals, who may be more threatening than low-status individuals^14^. Similarly, a greater gaze-cuing effect only emerged in other research on threatening faces when participants were presented with facial stimuli from both threatening and nonthreatening out-groups^15^. These findings suggest that humans may be vigilant for threatening stimuli or have more difficulty disengaging from threatening stimuli than from nonthreatening stimuli. One aim of the present study was to use a standard gaze-cuing paradigm to figure out the way how intergroup threat gates social attention in humans (based on the behavioral data). In other words, we want to investigate whether intergroup threatening cues draw attention faster or whether, once detected, intergroup-threat-related facial stimuli hold attention longer, or both.

The standard gaze-cuing paradigm can detect whether the gaze-cuing effect in a intergroup threat condition results from either attention vigilance or difficulty disengaging attention, or both, because the gaze-cueing task includes two critical types of trials: congruent and incongruent. On congruent trials, the target appears in the same location as the gaze cue. Because the gaze cue captures attention, which orients study participants to the location of the target, congruent trials should facilitate detection of the target. Critically, if threatening stimuli capture attention faster than nonthreatening stimuli, congruent trials in which a threatening cue appears should yield shorter reaction times (RTs) than congruent trials in which the nonthreatening cue appears, which indicates attention vigilance (facilitated attention) for intergroup threat. On incongruent trials, the target appears in the location opposite the gaze cue. Hence, participants must disengage from the gaze cue in order to detect the target, which requires more effort to inhibit than the congruent response requires. Because the incongruent cue holds attention, it should delay the response to the target. If threatening stimuli hold attention longer than nonthreatening stimuli do, incongruent trials in which threatening cues appear should yield longer RTs than incongruent trials in which nonthreatening cues appear, which reflects the difficulty of disengaging from an intergroup threat.

Notably, the underlying mechanisms also appear to be different from attention vigilance, as previous evidence suggests that attention vigilance involves a different neural network from difficulty in attention disengagement^16^. On one hand, research has found the amygdala is a central structure in a neural mechanism involved in attention vigilance (facilitated attention) for threat (a threat detect system)^17^,^18^,^19^,^20^. For example, previous research found that a patient with bilateral amygdala lesions did not detect threat information rapidly, whereas normal individuals with unilateral amygdala lesions did display vigilance to threat information^21^. On the other hand, a wealth of data are beginning to suggest that the activity of the prefrontal cortex (PFC) and its subunits and functionally-related structures – e.g., the anterior cingulate cortex (ACC) – are involved in the difficulty to disengage from threat^22^,^23^,^24^. Prefrontal regulatory structures, such as the inferior frontal gyrus, have been described as belonging to an attention control system^25^. Meanwhile, related research has found that individuals with good attention control can shift attention away from threatening information, whereas individuals with poor attention cannot do so^22^. Consequently, the other aim of the present study was to examine the neural basis of how intergroup threat gates social attention in humans (based on the imaging data). Specifically, we want to investigate whether intergroup threatening cues would activate a network which resembles the one that has been suggested to be involved in attention vigilance or a network involved in difficulty disengaging attention from intergroup threat, or both.

We modified the standard gaze-cuing paradigm^14^ for this purpose. In study *a*, intergroup threat between the Chinese (nonthreatening in-group) and Japanese (threatening out-group) was manipulated to generate a gaze-mediated intergroup threatening context. In study *b*, North Korean (nonthreatening out-group) was employed as nonthreatening out-group to generate a gaze-mediated nonthreatening context. Specially, Chinese facial stimuli were randomly divided into Chinese group and Japanese group in study *a* (Chinese group and North Korean group in study *b*), and the Chinese participants were asked to learn to which group each face belonged. This enabled our participants to distinguish clearly between the nonthreatening in-group faces and the threatening out-group faces in study *a* (nonthreatening in-group faces and the nonthreatening out-group faces in study *b*). Functional magnetic resonance imaging (fMRI) at 3T was performed while participants responded to a target that appeared to the left or right of the eye-gaze stimuli, which were either from threatening out-group or nonthreatening in-group in study *a* (which were either from nonthreatening out-group or nonthreatening in-group in study *b*). The direction of the shifted eye gaze can be either congruent or incongruent in the gaze-cuing paradigm, with the location of the target subsequently presented. Studies using this paradigm have shown that human observers tend to respond faster to targets presented in gaze-congruent locations than to targets presented in gaze-incongruent locations, a phenomenon that is often referred to as the gaze-cuing effect^6^,^7^,^26^,^27^. The stimulus onset asynchrony (SOA) used in this study was either 200 ms or 800 ms, as the involuntary component of the gaze-cuing effect is most likely to appear at a short SOA^6^,^15^,^28^, and it seems that the effects of gaze-cuing effects are stronger at shorter SOAs^29^, but a greater gaze-cuing effect also has been found to emerge at a long SOA (e.g. ≥800 ms or even lasted 3 minutes) in some studies^30^,^31^,^32^. Thus, our study used a 2 × 2 × 2 factorial design.

We conducted this study to examine the way and the neural basis of how intergroup threat gates social attention in humans. Firstly, our hypotheses were that either shorter RTs would emerge for threatening out-group faces compared to nonthreatening in-group faces in the congruent conditions, or longer RTs would emerge for threatening out-group faces compared to nonthreatening in-group faces in the incongruent conditions, or both situations would happen when a greater gaze-cuing effect showed for threatening out-group faces than for nonthreatening in-group faces in study *a*. Secondly, we also hypothesized that either the amygdala (a central structure in a neural mechanism involved in attention vigilance to threat) or the PFC (a central structure in a neural mechanism involved in the difficulty to disengage from threat), or both of these areas, would be activated when participants were shown threatening faces in study *a*. At last, no differences would emerge between nonthreatening out-group and nonthreatening in-group faces in study *b*.

## Results

### Behavioral data

#### Intergroup threat scores

The mean score on the intergroup threat question was 4.71 (*SD* = 1.15) and higher than the median (4) in study *a (t*(21) = 2.855, *p* = 0.010, *d* = 0.873), indicating that the participants felt the intergroup threat from Japan towards China. The mean score on the intergroup threat question was 2.53 (*SD* = 1.17) and lower than the median in study *b (t*(19) = 5.480, *p* < 0.001, *d* = 1.777), indicating that the participants did not feel the intergroup threat from North Korea towards China. Moreover, paired *t*-tests in study *a* showed that the post-test scores were higher than the pre-test scores for *worried* [*t*(21) = 3.189, *p* = 0.005, *d* = 0.982], *anxious* [*t*(21) = 3.873, *p* = 0.001, *d* = 1.001], *irritable* [*t*(21) = 6.473, *p* < 0.001, *d* = 1.705], *angry* [*t* (21) = 6.768, *p* < 0.001, *d* = 1.822], and *afraid* [ (21) = 2.911, *p* = 0.009, *d* = 0.633]. However, there was no difference between pre-test scores and post-test scores for any emotion in study *b* (all *p*’s > 0.104). Those results confirmed that the manipulation in the present study was successful.

#### Reaction times

All the trials with incorrect responses were discarded (*a:* 1.29%, *b*: 3.25%). A 2 (cue-target spatial congruency: congruent vs. incongruent) × 2 (intergroup threat: threatening out-group vs. nonthreatening in-group in study *a*; nonthreatening groups: nonthreatening out-group vs. nonthreatening in-group in study *b*) × 2 (SOA: 200 ms vs. 800 ms) repeated-measures analysis of variance (ANOVA) was performed on the mean RTs. In study *a*, the analysis found significant main effects of cue-target spatial congruency [*F*(1, 20) = 10.471, *p* = 0.004, partial *η*^2^ = 0.344] and SOA [*F*(1, 20) = 30.922, *p* < 0.001, partial *η*^2^ = 0.607], and a significant three-way interaction [*F*(1, 20) = 7.964, *p* = 0.011, partial *η*^2^ = 0.285]. However, the main effect of threat [*F*(1, 20) = 0.660, *p* = 0.426, partial *η*^2^ = 0.032] and the two-way interactions were not significant [all *F*s < 1.362, all *p*s > 0.257, and all partial *η*^2^s < 0.064]. In study *b*, the main effects of cue-target spatial congruency [*F*(1, 18) = 8.567, *p* = 0.009, partial *η*^2^ = 0.322] and SOA were significant [*F*(1, 18) = 39.715, *p* < 0.001, partial *η*^2^ = 0.688]. No other significant effect emerged (all *p*’s > 0.131).

**Figure 1:**
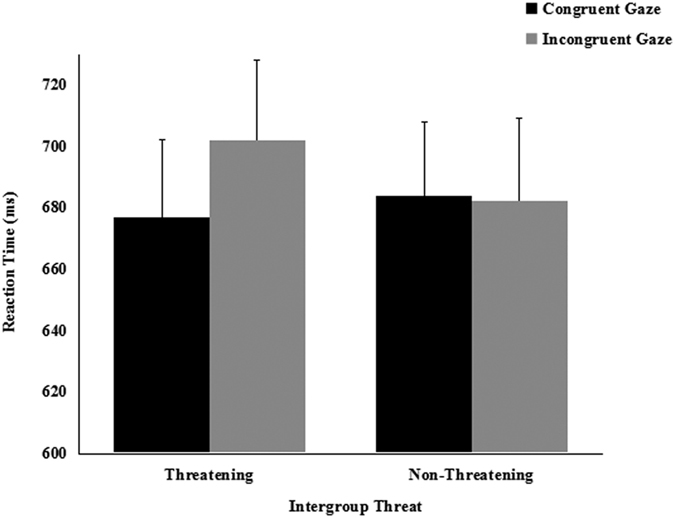
Mean reaction times (±*SE*) as a function of intergroup threat and cue congruency for 200-ms SOA in study *a*.

**Figure 2:**
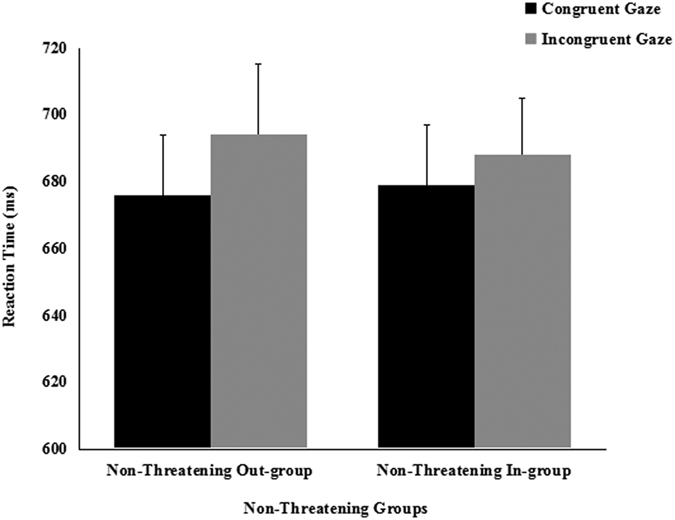
Mean reaction times (±*SE*) as a function of nonthreatening groups and cue congruency for 200-ms SOA in study *b*.

A 2 (cue-target spatial congruent: congruent vs. incongruent) × 2 (threat: threatening vs. nonthreatening) repeated-measures ANVOA was conducted separately on the mean RTs in the 200 ms and the 800 ms SOA conditions. In study *a*, the main effect of the spatial congruency of the cue-target in the 200 ms SOA condition approached statistical significance [*F*(1, 20) = 3.953, *p* = 0.061, partial *η*^2^ = 0.165], whereas the main effect of threat was not significant [*F*(1, 20) = 1.294, *p* = 0.269, partial *η*^2^ = 0.061]. The interaction of spatial congruency × threat was significant [*F*(1, 20) = 9.816, *p* = 0.005, partial *η*^2^ = 0.329]. Paired *t*-tests used to interpret the interaction, found that participants shifted their attention in response to the averted gaze of the threatening out-group faces [*t* (21) = 3.242, *p* = 0.004, *d* = 0.216], but they did not shift attention in response to the averted gaze of the nonthreatening in-group faces [*t* (21) = 0.495, *p* = 0.626, *d* = 0.026] (Fig. 1). Interestingly, it took participants in the congruent condition almost the same time to follow the gaze of threatening out-group faces as the nonthreatening in-group faces [*t* (21) = 1.092, *p* = 0.288, *d* = 0.063]. However, it took participants in the incongruent condition more time (longer RTs) to suppress the influence of the gaze direction of the threatening out-group faces, compared to the nonthreatening in-group faces [*t* (21) = 2.846, *p* = 0.010, *d* = 0.166]. In study *b*, we only find a significant main effect of cue-target spatial congruency [*F*(1, 18) = 4.641, *p* = 0.045, partial *η*^2^ = 0.205], the main effect of groups[*F*(1, 18) = 0.095, *p* = 0.761, partial *η*^2^ = 0.005] and the two-way interaction[*F*(1, 18) = 0.654, *p* = 0.429, partial *η*^2^ = 0.035] were not significant. Paired *t*-tests found that participants did not shift their attention in response to the averted gaze of the nonthreatening out-group faces [*t* (19) = 1.756, *p* = 0.096, *d* = 0.223] or the nonthreatening in-group faces [*t* (19) = 1.055, *p* = 0.305, *d* = 0.101] (Fig. 2). It took participants almost the same time to follow the gaze of nonthreatening out-group faces as the nonthreatening in-group faces in both the congruent condition [*t* (19) = 0.538, *p* = 0.597, *d* = 0.050] and the incongruent condition [*t* (19) = 0.791, *p* = 0.439, *d* = 0.030].

**Figure 3:**
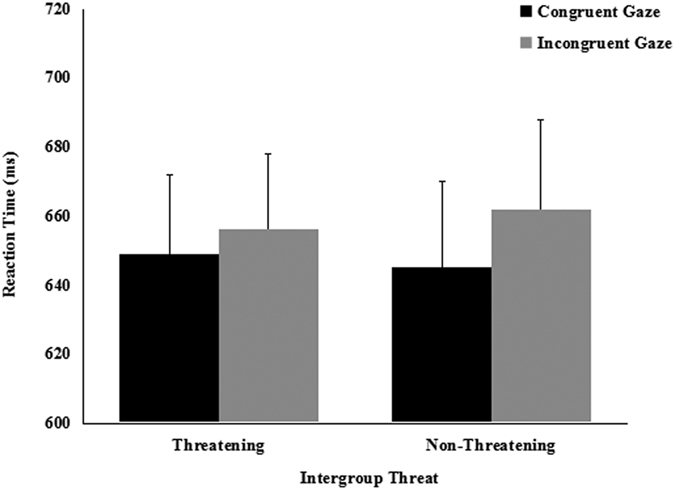
Mean reaction times (±*SE*) as a function of intergroup threat and cue congruency for 800-ms SOA in study *a*.

**Figure 4:**
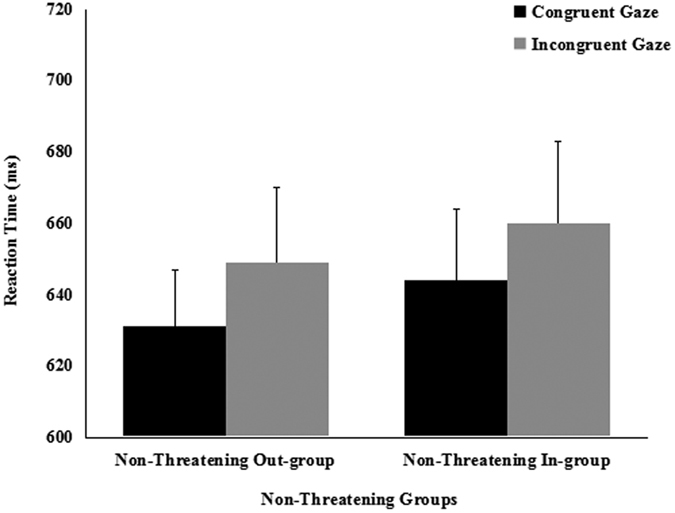
Mean reaction times (±*SE*) as a function of nonthreatening groups and cue congruency for 800-ms SOA in study *b*.

The same ANOVA was conducted for the 800 ms condition SOA. In study *a*, we found a significant main effect of cue-target spatial congruency [*F*(1, 20) = 6.219, *p* = 0.022, partial *η*^2^ = 0.237), due to faster RTs on congruent (*M* = 647 ms, *SE* = 22.47) than on incongruent (*M* = 659 ms, *SE* = 23.43) trials. The main effect of threat [*F*(1, 20) = 0.046, *p* = 0.832, partial *η*^2^ = 0.002), and the spatial congruence × threat interaction [*F*(1, 20) = 0.662, *p* = 0.426, partial *η*^2^ = 0.032] were not significant. Paired *t*-tests found that participants did not shift their attention in response to the averted gaze of the threatening out-group faces [*t* (21) = 1.125, *p* = 0.274, *d* = 0.067] or to the averted gaze of the nonthreatening in-group faces [*t* (21) = 1.999, *p* = 0.059, *d* = 0.156] (Fig. 3). It took the participants in the congruent condition almost the same time to follow the gaze of the threatening out-group faces and the nonthreatening in-group faces [*t* (21) = 0.590, *p* = 0.562, *d* = 0.038]. It also took participants in the incongruent condition the same time to suppress the gaze of the threatening out-group and nonthreatening in-group faces [*t* (21) = 0.674, *p* = 0.508, *d* = 0.055]. In study *b*, we only find a significant main effect of cue-target spatial congruency [*F*(1, 18) = 5.205, *p* = 0.035, partial *η*^2^ = 0.224], the main effect of groups[*F*(1, 18) = 3.968, *p* = 0.062, partial *η*^2^ = 0.181] and the two-way interaction[*F*(1, 18) = 0.094, *p* = 0.762, partial *η*^2^ = 0.005] were not significant. Paired *t*-tests found that participants did not shift their attention in response to the averted gaze of the nonthreatening out-group faces [*t* (19) = 2.069, *p* = 0.053, *d* = 0.219] or the nonthreatening in-group faces [*t* (19) = 1.749, *p* = 0.097, *d* = 0.157] (Fig. 4). It took participants almost the same time to follow the gaze of nonthreatening out-group faces as the nonthreatening in-group faces in both the congruent condition [*t* (19) = 1.669, *p* = 0.112, *d* = 0.145] and the incongruent condition [*t* (19) = 1.416, *p* = 0.174, *d* = 0.102].

#### Error rates

The same 2 × 2 × 2 ANVOA conducted on the RT data was used to analyze the percentage of errors. In study *a*, the ANOVA revealed a significant main effect of SOA [*F*(1, 20) = 8.559, *p* = 0.008, partial *η*^2^ = 0.300), indicating that participants made more errors when the SOA was 200 ms (*M* = 1.7%, *SE* = 0.005) than when the SOA was 800 ms (*M* = 0.7%, *SE* = 0.002). The main effect of threat approached statistical significance [*F* (1, 20) = 4.316, *p* = 0.051, partial *η*^2^ = 0.177], reflecting the fact that participants tended to commit fewer errors when they observed nonthreatening faces (*M* = 1%, *SE* = 0.004) than when observed threatening faces (*M* = 1.5%, *SE* = 0.004). No other significant effects emerged [all *F*s < 2.543, all *p*s > 0.126, and all partial *η*^2^s < 0.113]. In study *b*, we did not find any significant effect (all *p*’s > 0.139). Thus, a speed-accuracy trade-off cannot account for the present findings (see the supplementary information).

### fMRI

#### Full factorial analysis

**Figure 5:**
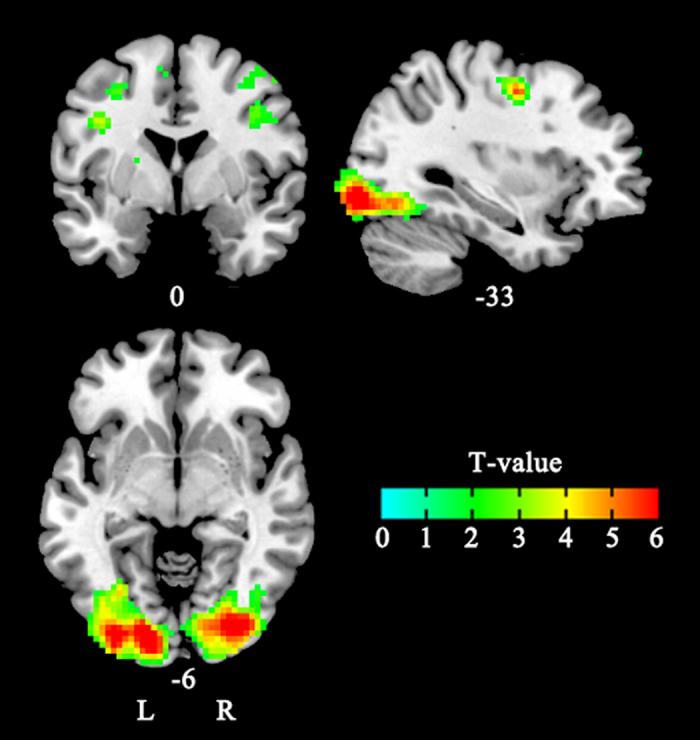
Maps showing brain areas in which activity was greater for nonthreatening in-group faces than for threatening out-group faces in study *a* (in the condition of congruent at 200 SOA) (FDR = 0.05, corrected).

**Figure 6:**
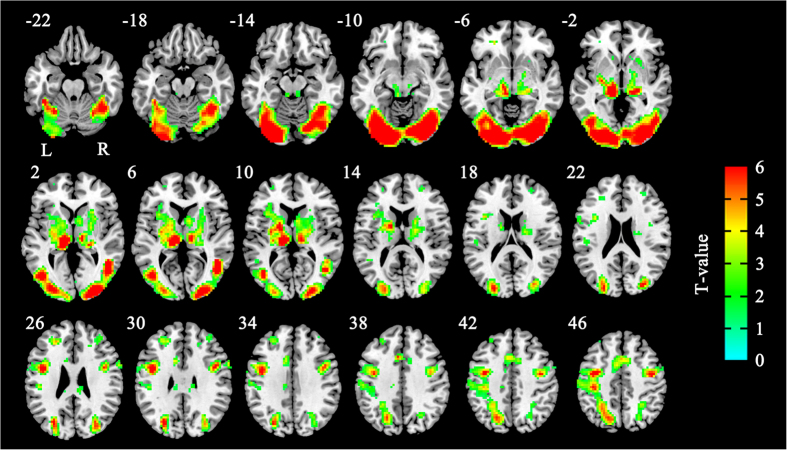
Maps showing brain areas in which activity was greater for threatening out-group faces than for nonthreatening in-group faces in study *a* (in the condition of incongruent at 200 SOA) (FDR = 0.05, corrected).

In study *a,* no suprathreshold voxels were found for neural correlates of any of the main effects or their interactions. Interestingly, suprathreshold voxels of activation only emerged in two conditions at the 200 ms SOA, but not at the 800 ms SOA, which is consistent with our behavioral data. We further examined the neural components involved in processing intergroup threat by comparing brain activity in response to threatening and nonthreatening faces. In the congruent conditions at 200 ms SOA, nonthreatening gaze-averted faces were associated with greater activity in the visual cortex than were threatening faces (see Table 1, Fig. 5); no regions were more active in response to nonthreatening faces than in response to threatening faces. What is more important is that, in the incongruent conditions at the 200 ms SOA, threatening gaze-averted faces were associated with greater activity in the attention and visual cortex than were nonthreatening faces (see Table 2, Fig. 6); no regions were more active in response to nonthreatening faces than in response to threatening faces. In study *b*, no suprathreshold voxels of activation was found, which is also consistent with our behavioral data.

#### Correlation analyses

We tested whether the greater neural activity in response to viewing a threatening face in the 200 ms SOA condition in study *a*, relative to a nonthreatening in-group face, was associated with individual differences in the perception of intergroup threat (e.g., feeling more anger after the intergroup threat manipulation). We calculated individual differences in brain activity for each ROI in the two conditions at the 200 ms SOA when the participants watched threatening out-group gazes, and then correlated these scores with individual differences in self-reported perceptions of intergroup threat (the intergroup threat score and the five emotion scores). No significant correlation emerged when participants watched threatening gazes in the congruent condition at the 200 ms SOA. In contrast, participants in the incongruent condition at the 200 ms SOA who felt greater anger showed stronger activity in the left mPFC (*r* = 0.541, *p* = 0.011), right caudate (*r* = 0.455, *p* = 0.038), left thalamus (*r* = 0.448, *p* = 0.042) and the right thalamus (*r* = 0.479, *p* = 0.028) when they were shown the gazes of threatening faces (FDR correction). No other significant correlation was found.

## Discussion

We used fMRI in this study to investigate the effect of intergroup threat on the neural basis of social attention, as assessed by a gaze-cuing task. In study *a,* the behavioral data showed that a greater gaze-cuing effect only emerged for threatening cues at the 200 ms SOA in the gaze-cuing task (a spatial orienting task). Interestingly, in the congruent condition(study *a*), there was no difference between the RTs for the intergroup threatening gaze or the nonthreatening gaze, which means intergroup threatening information did not draw attention faster than did nonthreatening information. However, it took participants more time (longer RTs) to disengage from the gaze direction of a threatening out-group face than a nonthreatening in-group face in the incongruent condition, which suggests that the intergroup threatening gaze did hold attention longer than did the nonthreatening gaze. The imaging data (study *a*) suggested that attention holding by threatening cues in the incongruent trials was supported by attention control (difficulty to disengage from threat) networks only at the 200 ms SOA. Moreover, the activity of the mPFC, caudate, and the thalamus correlated with self-reported anger in response to the intergroup threat manipulation. Moreover, no significant difference emerged between nonthreatening out-group faces and nonthreatening in-group faces for both SOAs in study *b*, which could rule out some alternative hypotheses that could explain our results (e.g., group membership, etc.). Taken together, our results suggest that intergroup threat would gate social attention by activating a network, which may resemble that suggested to be involved in the involuntary holding of attention (difficulty disengaging from a threat) rather than drawing attention (vigilance or facilitated attention to detect threat).

In study *a*, participants only shifted their attention in response to the averted gaze of threatening out-group rather than nonthreatening in-group faces. In study *b*, we found that participants did not shifted their attention in response to the averted gaze of nonthreatening out-group faces or nonthreatening in-group faces. These findings suggest that only when human beings felt the sense of intergroup threat from an out-group, could they shift attention with the gaze direction of the faces of that out-group, which is consistent with our previous research^15^. Based on those results, we believe that intergroup threat is a moderator of gaze cuing and plays an important role in shaping social attention. It should be point out that, meanwhile, larger gaze cuing effect for threatening than nonthreatening faces only emerged at 200-ms SOA in study *a*, the specificity of the effect at the short SOA suggests that intergroup threat cues modulate the involuntary component of gaze-cuing, which is also consistent with the previous work demonstrating threat-contingent gaze-cuing^15^,^30^.

The ability to respond to threatening stimuli quickly and validly is crucial for the survival of both an individual and a group^33^. We only found difficulty disengaging from a threatening gaze in the intergroup threat condition, which is consistent with previous studies. Researchers have observed that difficulty disengaging from a threat is independent of vigilance (i.e., facilitated attention) to threat, but when vigilance occurs difficulty with disengagement also emerges^34^,^35^,^36^,^37^. Threat-related studies provide evidence that attention can be captured by threatening stimuli, such as snakes and angry faces^38^,^39^, which can be interpreted as vigilance for threat. Previous studies also have suggested that the facilitated response to probes at the threat location may arise from difficulty disengaging from the threat location rather than from vigilance for threat^22^,^40^. Furthermore, studies using an emotional variants of the exogenous cueing task have revealed that anxious individuals are not characterized by vigilance for threat (facilitated engagement), but by difficulty disengaging attention from threat^34^,^41^. These findings suggest that disengagement may be more important than vigilance for reacting to threat in the gaze-cuing effect in the gaze-cuing task, just like our findings in this study.

This study specifically investigated the notion that introducing threatening gaze cues into an established gaze-cuing paradigm would allow us to investigate the effect of threat in an intergroup context on the neural correlates of attention bias. Here, we expected that intergroup threat would gate social attention by activating either a network which would resemble that suggested to be involved in attention vigilance or difficulty disengaging attention from intergroup threat, or both. In line with this hypothesis, we found that intergroup threatening cues altered the neural network described above that subserve attention control, including brain areas that have been implicated in difficulty disengaging from intergroup threat in the 200 ms SOA^42^,^43^. We did not find any brain area activity during the congruent trials – not even in the amygdala, which is described as a central structure of a threat detection system. The fMRI data for the incongruent condition at the 200 ms SOA revealed that incongruent responses to intergroup threatening cues, compared to nonthreatening cues, increased neural activity in the inferior, middle, and superior frontal gyrus, the thalamus, fusiform gyrus, dorsal anterior cingulate cortex, putamen, and the right caudate. These findings are consistent with our behavioral results and provide support for the idea that intergroup threat gates social attention by holding social attention longer, rather than capturing attention faster.

As is the case of the amygdala, the PFC, especially the inferior frontal cortex, is conceptualized as a critical structure in an attention control system that includes functionally-related structures (e.g., ACC) of the PFC^16^. This control mechanism may be responsible for the difficulty in disengaging from threat^16^. On one hand, numerous studies have demonstrated activation of the inferior frontal cortex during the processing of response inhibition and cognitive control^43^,^44^,^45^,^46^,^47^. Similarly, evidence suggests that the inferior frontal gyrus can be engaged during inhibitory control^48^,^49^. On the other hand, the early processing of threat information is fast and automatic^33^,^50^, and a threatening stimulus is likely to have a more pronounced effect on preferential processing, so the gaze shifts of a threatening face in the context of intergroup threat might trigger imitative responses that require a more pronounced inhibitory effort^51^,^52^,^53^. With respect to our paradigm, it makes sense to assume that the generation of an incongruent response to an intergroup threatening face constitutes such a situation.

Apart from cognitive control and response inhibition, the inferior frontal cortex has been described as belonging to a “ventral attention network” (VAN), whose activity may contribute to reorienting attention and allowing a reorienting response^42^. Activity in the VAN may direct cognition and contribute to reorienting from one task state to another, so that stimuli can be linked to gaze following responses^45^. The intergroup threatening stimuli in the present study specifically engaged the inferior frontal cortex of the VAN, which may have resulted from threatening stimuli imposing additional constraints on performing an incongruent reaction. Threatening gaze shifts can be assumed to affect spatial orienting, and they are known to be perceived as indicative of potentially relevant threat information in the environment^2^,^54^,^55^. Therefore, we can tentatively suggest that the generation of an incongruent reaction to the gaze of a threatening face may lead to increased inhibitory processes, which are necessary to disengage from imitative reactions. At the same time, inhibiting the urge to orient in the direction of the gaze shift of a threatening face may lead to more cognitive activity that is related to processing the threatening stimulus in terms of its underlying meaning.

In parallel with the above-described findings, an increase in neural activity was observed in the thalamus and the dACC, which have been suggested to be involved in the regulation of “executive functions”^56^. Importantly, there also is evidence for anatomical and functional connectivity between the inferior frontal cortex, the thalamus, and the basal ganglia, which were concomitantly activated in our study, indicating that they constitute a “cognitive control network”^57^,^58^,^59^. We suggest that the modulation of gaze cuing processes might be particularly relevant in the case of difficulty disengaging from threatening stimuli, which would account for the co-activation of the inferior frontal gyrus, the thalamus, the dACC, and the caudate. Furthermore, anger, which is different from the other four emotions in the study, is often considered be one of the basic emotions, and it is the common threat-related emotion that is elicited when people confront survival-related problems^60^. This might be the reason why the activity of the mPFC, caudate, and thalamus were only associated with self-reported anger after the intergroup threat manipulation in the present study.

## Methods

### Participants

Twenty-one right-handed Chinese students (12 females) from Southwest University with normal or corrected-to-normal vision participated in the study *a*. Nineteen right-handed Chinese students (11 females) from Southwest University with normal or corrected-to-normal vision participated in the study *b*. They were between the ages of 19 and 27 (*a: M* = 22.33 years, *SD* = 1.88, *b: M* = 21.26 years, *SD* = 2.13), and they did not report any history of neurologic or psychiatric illness. All participants provided written informed consent, and the ethical standards in conducting the research are in line with the Declaration of Helsinki and were granted approval by the Researcher’s Review Board of Southwest University.

### Stimuli and Procedures

Eight black-and-white photos of neutral faces from the Chinese Facial Affective Picture System (CFAPS)^61^ were used as stimuli (4 males and 4 females), which were randomly divided into two groups; one group of photos was classified as Chinese (2 males and 2 females) and the other group was classified as Japanese (2 males and 2 females) in study *a*, one group of photos was classified as Chinese (2 males and 2 females) and the other group was classified as North korean (2 males and 2 females) in study *b*.

The participants completed a brief questionnaire that measured their feelings on 5 emotions as soon as they arrived at the laboratory, which served as a pre-test. The participants rated each emotion on a 5-point scale ranging from 1 (“a little”) to 5 (“quite a lot”). The 5 emotions were *worried, anxious, irritable, angry,* and *afraid,* which previous studies have found to be related to a sense of threat^60^. Then, the participants were given three minutes to learn which country was associated with each photo. To make sure that participants had learned the association between each county and each photo, each photo was presented 3 times for a total of 24 recognition trails. The participants were given feedback that their response was “CORRECT” or “WRONG” on each response. The correct response rate had to be over 95%.

The participants in study *a* read an article about the Sino-Japanese War that contained 3 pictures, participants in study *b* read an article about the Sino-Korean nonthreatening history that contained 3 pictures. Afterward, the measure of emotion was completed again by participants as a post-test to check the effectiveness of the intergroup threat/nonthreatening condition manipulation. Then, they were asked one more question: “How much intergroup threat do you feel from Japan/North Korea toward China after reading the article and observing the pictures?” (rated from 1 = “not at all” to 7 = “very much”). Following the intergroup threat/nonthreatening condition manipulation check, a gaze-cuing task was completed by all the participants.

**Figure 7:**
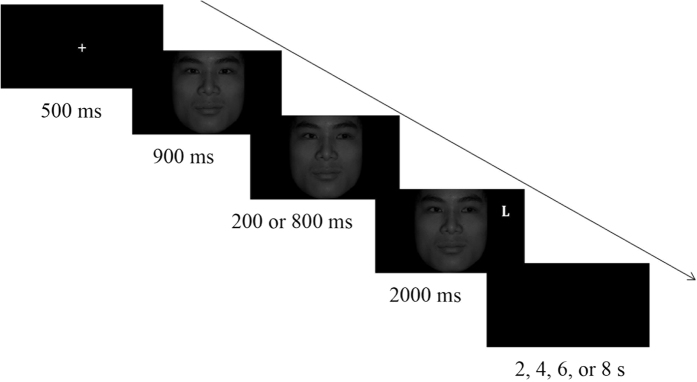
Schematic illustration of gaze-cuing task. The figure shows an example of a single trial. The direction of the male gaze was informative as to the location of the letter “L” target.

An improved gaze-cuing task was used in the present study based on a study by Dalmaso *et al*.^14^. A white cross was shown for 500 ms at the beginning of the task, which was followed by a face stimulus that was presented with a direct gaze for 900 ms. Then, the pupils of the face moved to the left or right for 200 ms or 800 ms, and a subsequent white target (threatening “T” or nonthreatening “L”) appeared for 2000 ms. Finally, a black screen appeared for 2 s, 4 s, 6 s, or 8 s (Fig. 7).

Response speed and accuracy were emphasized in the instructions; the participants were instructed to respond as quickly and accurately as possible, and to ignore the shifted gaze, as gaze cues did not predict the probable location of the target. Participants responded by pressing the “1” or “2” key with their right index finger. Half of the participants pressed the “1” key if the target was a “T,” and they pressed “2” key if the target was an “L.” The other half of the participants made the opposite response to each target. The trials were split into four blocks of 64 trails, which were fully counterbalanced in terms of cue-target spatial congruency (congruent, incongruent), intergroup threat (threatening, nonthreatening), and SOA (200 ms, 800 ms). Thus, there were 256 trials in total and the order of the trials was pseudo-random within each block. Each participant completed 24 practice trials before the experimental trials.

### Behavioral data analysis

The reaction times (RTs) and the percentage of correct responses (CRs) were the dependent variables. The behavioral measurements obtained during the fMRI experiment were analyzed off-line using SPSS 16.0. Errors and outlier latencies greater than 1500 ms or less than 100 ms were removed as missed responses or anticipation errors (1.29% of all responses). The effects of the experimental factors (response type: “congruent vs. incongruent”; stimulus type: “threatening vs. nonthreatening”; SOA: 200 ms vs. 800 ms) on mean RT and the CR percentage were compared using repeated-measures analysis of variance (ANOVA).

### MRI data acquisition

Scanning was performed with a 3.0 T Siemens Trio system using a standard head coil at the Southwest University MRI Center for Brain Research. Anatomical images were acquired using a standard 3D T1-weighted sequence (1 × 1 × 1 mm^3^ high-resolution). Functional images that covered the whole brain were done with an EPI sequence (TR = 2000 ms, TE = 30 ms, FOV = 220 mm, flip angle = 90°, matrix = 64 × 64, slice thickness = 3.0 mm, 32 slices, interleaved slice mode, and voxel size = 3.4 × 3.4 × 3 mm^3^).

### Imaging preprocessing

Data preprocessing was performed using Statistical Parametric Mapping software (SPM8; http://www.fil.ion.ucl.ac.uk/spm/software/spm8/). The first five volumes were discarded to allow for T1 equilibrium effects. The remaining volumes were realigned to compensate for small head movements^62^. The movement threshold for translation was set at 2 mm and the rotational movement threshold was set at 2^◦^. Data were spatially normalized to a standard template (Montreal Neurological Institute) with resampling to 3 mm × 3 mm × 3 mm. Spatial smoothing of the data was done using a 8 mm full-width-half-maximum Gaussian kernel. The data were filtered in the temporal domain using a nonlinear high-pass filter with a 128 s cutoff.

### First-level analysis

The following events were modeled after convolution with a canonical hemodynamic response function (HRF): intergroup threat (THR vs. NON in study *a*, NON-IN vs. NON-OUT in study *b*), response type (CON vs. INC), and SOA (200 ms vs. 800 ms). The head movement parameters were included as covariates of no interest to improve statistical sensitivity.

### Second-level analysis

A higher-level analysis created across-session contrasts for each participant for a set of contrast images. These were then analyzed at the whole-brain level, with random-effects analyses in SPM8, using a full factorial analysis (factor: intergroup threat, response type, SOA). Group images were thresholded using the false discovery rate (FDR) correction (*P* < 0.05), corrected for whole-brain multiple comparisons.

Further analyses used regions of interest (ROIs) to explore the brain correlations of attention vigilance under the intergroup threat condition, for each congruent condition. Meanwhile, ROIs were used to investigate the brain correlates of difficulty disengaging attention under the intergroup threat condition for each incongruent condition. We selected the significantly different regions as ROIs that had a small 6 mm centered sphere as seen in the center coordinates in Tables 1 and 2. Then, we extracted the percent signal change of each ROI in each condition to perform correlation analyses with intergroup threat scores and the five emotional measures (posttest-scores minus pretest-scores). The results were thresholded at *P* < 0.05, and these *p*-values were adjusted with FDR correction.

## Conclusions

In summary, the present study provides evidence that the automatic orienting of visual attention in the context of intergroup threat involves a mechanism when shifts of attention are held by incongruent threatening gaze cues (i.e., difficulty disengaging attention from an intergroup threat) rather than by attention being drawn to congruent threatening cues (i.e., attention vigilance to intergroup threat). These results provide support for the notion that an intergroup threatening gaze by a person may, indeed, be a special stimulus for holding social attention.

## Additional Information

**How to cite this article**: Chen, Y. *et al*. The neural basis of intergroup threat effect on social attention. *Sci. Rep.* **7**, 41062; doi: 10.1038/srep41062 (2017).

**Publisher's note:** Springer Nature remains neutral with regard to jurisdictional claims in published maps and institutional affiliations.

## Supplementary Material

Supplementary Tables

## Figures and Tables

**Table 1:** Regions of significantly greater activity in response to nonthreatening faces relative to threatening faces (nonthreatening > threatening) in the congruent condition at the 200 ms SOA (in study *a*)*.

Anatomical region	MNI Coordinates			*t*-value	Cluster Size
	x	y	z		
Left supplementary motor area	−6	6	54	4.31	46
Left Calcarine gyrus	−12	−90	−6	6.86	70
Left fusiform gyrus	−21	−84	−6	6.27	141
Right fusiform gyrus	27	−81	−9	6.68	223
Left lingual gyrus	−18	−84	−6	6.95	129
Right lingual gyrus	24	−81	−9	6.64	168
Left middle occipital gyrus	−18	−87	−6	7.19	250
Right middle occipital gyrus	30	−87	0	5.08	98
Left inferior occipital gyrus	−33	−87	−9	6.89	227
Right inferior occipital gyrus	33	−81	−6	6.81	158
Left precentral gyrus	−33	−6	48	5.69	125
Right precentral gyrus	45	6	30	4.58	74

^*^FDR = 0.05, corrected.

**Table 2:** Regions of significantly greater activity in response to threatening faces relative to nonthreatening faces (threatening > nonthreatening) in the incongruent condition at the 200 ms SOA (in study *a*)*.

Anatomical region	MNI Coordinates			*t*-value	Cluster Size
	x	y	z		
Lef opercular part of inferior frontal gyrus	−39	3	27	5.05	75
Left middle frontal gyrus	−30	−6	51	5.45	127
Right middle frontal gyrus	45	−3	54	6.41	172
Right dorsolateral frontal gyrus	36	−6	66	4.20	81
Left fusiform gyrus	−21	−84	−9	8.90	356
Right fusiform gyrus	30	−84	−6	8.53	392
Left Insula	−33	15	6	3.77	72
Left lingual gyrus	−12	−90	−9	9.64	249
Right lingual gyrus	24	−84	−6	7.77	269
Left inferior occipital gyrus	−18	−93	−9	9.45	299
Right inferior occipital gyrus	30	−87	−6	8.33	225
Left middle occipital gyrus	−15	−90	−6	10.95	678
Right middle occipital gyrus	27	−90	0	6.86	343
Left superior occipital gyrus	−24	−72	33	4.71	142
Right superior occipital gyrus	21	−93	6	5.67	137
Left inferior parietal gyrus	−24	−54	54	5.08	206
Left superior parietal gyrus	−24	−57	48	5.14	225
Right superior parietal gyrus	24	−57	54	4.11	74
Left postcentral gyrus	−54	−18	54	5.84	560
Left precentral gyrus	−39	−6	48	6.53	733
Right precentral gyrus	42	−6	48	6.79	351
Left Putamen	−18	0	9	5.20	190
Right Putamen	24	−6	9	4.36	116
Left supplementary motor area	−6	6	7	6.18	287
Right supplementary motor area	6	12	51	6.08	204
Right inferior tempotal gyrus	45	−69	−9	5.91	204
Left inferior tempotal gyrus	−42	−66	9	5.50	110
Right middle temporal gyrus	48	−63	3	6.95	180
Left Thalamus	−9	−21	3	8.13	256
Right Thalamus	21	−27	0	5.91	199
Left Calcarine gyrus	−12	−90	−6	11.25	173
Right Calcarine gyrus	24	−93	0	6.79	124
Right Caudate	18	6	15	4.15	68
Left dorsal anterior cingulate cortex	−6	15	39	4.43	78
Right dorsal anterior cingulate cortex	9	12	42	3.58	35

^*^FDR = 0.05, corrected.
