# Supplementary material for: The neural basis of intergroup threat effect on social attention
Source: Sci Rep. 2017 Jan 25;7:41062. doi: 10.1038/srep41062 (PMC5264403; doi:10.1038/srep41062)
Supplement: Supplementary Tables [file srep41062-s1.pdf]

The neural basis of intergroup threat effect on social attention

Yujie Chen[1, 2], Yufang Zhao[1, 2*], Hongwen Song[3], Lili Guan[4], Xin Wu[1, 2]

[1]School of Psychology, Southwest University, Chongqing, People's Republic of China

[2]Key Laboratory of Cognition and Personality, Ministry of Education, Chongqing, People's Republic of China

[3]School of Humanities and Social Science, University of Science and Technology of China, Anhui, People's Republic of China

[4]School of Psychology, Northeast Normal University, Changchun, People's Republic of China

Please address correspondence to:

Yufang Zhao

School of Psychology, Southwest University,

No. 2, TianSheng RD., Beibei, ChongQing 400715, China

TEL: +86 23 68367895

E-mail: zhaobee@swu.edu.cn

Table_S1. Data of emotion measure (5-point scale) and the sense of intergroup threat in study *a*.

ID	gender	age	pre_ worried	pre_ anxious	pre_ irritable	pre_ angry	pre_ afraid	post_ worried	post_ anxious	post_ irritable	post_ angry	post_ afraid	score_ threat
1	1	23	2	1	1	1	2	3	3	1	1	1	5
2	2	25	1	1	1	1	1	3	1	1	3	2	5
3	1	20	2	2	1	1	1	3	3	3	4	4	6
4	1	23	2	1	1	1	1	1	3	4	3	3	1
5	1	19	2	1	1	1	1	3	4	4	4	4	5
6	2	19	1	1	1	1	1	2	3	4	3	4	5
7	1	22	2	1	1	1	1	2	1	2	4	4	4
8	1	22	1	1	1	1	1	4	4	3	5	5	4
9	2	23	2	1	1	1	1	2	2	4	4	3	5
10	2	25	1	2	3	3	1	3	3	5	5	5	5
11	1	24	1	2	1	1	2	5	3	4	5	5	5
12	1	24	4	4	4	3	3	4	4	4	4	4	6
13	2	22	1	1	1	1	1	1	1	3	1	2	4
14	1	19	1	1	1	1	1	3	3	4	4	4	6
15	1	23	1	1	1	1	1	2	1	1	1	1	4
16	2	24	1	1	1	1	1	2	1	1	2	1	6
17	2	24	2	1	1	1	1	1	1	2	2	2	4
18	2	21	1	1	1	1	1	2	1	1	2	2	4
19	1	21	2	2	1	1	2	2	3	2	2	3	6
20	1	23	1	1	1	1	1	3	4	2	4	5	5
21	2	23	2	2	1	1	1	1	1	1	2	2	4

Note:

"ID" means "identification of participants";

"gender": "1" means "female"; "2" means "male";

"pre_worried" means "pretest score of worried emotion"

"pre_ anxious" means "pretest score of anxious emotion"

"pre_ angry" means "pretest score of angry emotion"

"pre_ irritable" means "pretest score of irritable emotion"

"pre_ afraid" means "pretest score of afraid emotion"

"post _worried" means "posttest score of worried emotion"

"post _ anxious" means "posttest score of anxious emotion"

"post _ angry" means "posttest score of angry emotion"

"post _ irritable" means "posttest score of irritable emotion"

“post _ afraid” means “posttest score of afraid emotion”

“score_ threat” means “the score of the sense of intergroup threat”.

Table_S2. Mean Reaction time in the gaze-cuing task in study *a*.

ID	SOA_200_ con _ nonthreatening (ms)	SOA_200_ incon _ nonthreatening (ms)	SOA_200_ con _ threatening (ms)	SOA_200_ incon _ threatening (ms)	SOA_800_ con _ nonthreatening (ms)	SOA_800_ incon _ nonthreatening (ms)	SOA_800- con _ threatening (ms)	SOA_800_ incon _ threatening (ms)
1	858.84	836.91	785.66	820.97	759.94	776.16	775.22	810.16
2	823.69	816.63	760.38	796.56	756.84	737.63	742.78	750.13
3	693.47	675.29	692.09	718.38	592.66	615.69	606.94	606.06
4	776.59	821.78	774.31	808.45	700.28	738.38	694.09	739.66
5	804.31	881.29	872.69	972.81	840.91	920.47	907.22	837.5
6	610.61	611.22	605.97	619.25	600.03	635.97	563.88	627.53
7	892.3	880.71	865.14	903.17	790.32	877.32	818.73	812.26
8	624.28	624.31	625.78	652.44	606.66	621.19	631.72	597.22
9	673.56	667.34	674.71	664.56	619.53	622.5	606.22	636.03
10	626.77	641.19	622.26	704.76	575.31	614.71	596.9	609.74
11	540.88	529.84	555.77	545.42	507.13	528.22	507.71	526.34
12	615.06	568.9	560	594.63	615.88	564.13	620.91	652.28
13	564.56	562.28	552.83	564.09	508.94	522.22	508	516.84
14	532.84	532.39	533.06	537.29	529.03	521.56	512.59	523.07
15	749.09	737.47	776.81	711.25	678.88	721.06	701.75	743.59
16	651.1	628.78	633.33	701.31	628.63	631.25	630.73	637.03
17	845.31	837.41	837.13	874.09	775.56	840.88	762.47	745.47
18	557	596.94	569.97	579.9	547.84	543.47	568.63	549.03
19	622.72	580.71	614.31	640.03	565.19	606.84	600.09	623.74
20	574.53	586.94	608.87	591.84	573.41	576.22	576.06	542.22
21	744.38	695.91	695.23	747.72	766.41	685	686.16	692.06

Note:

“SOA_200_con _ nonthreatening” means “mean RT of trails where non-threatening in-group faces’ gaze direction and target location were congruent at the 200 ms SOA”;

“SOA_200_incon_nonthreatening” means “mean RT of trails where non-threatening in-group faces’ gaze direction and target location were incongruent at the 200 ms SOA”;

“SOA_200_con_threatening” means “mean RT of trails where threatening out-group faces’ gaze direction and target location were congruent at the 200 ms SOA”;

“SOA_200_incon _threatening” means “mean RT of trails where threatening out-group faces’ gaze direction and target location were incongruent at the 200 ms SOA”;

“SOA_800_con _ nonthreatening” means “mean RT of trails where non-threatening in-group faces’ gaze direction and target location were congruent at the 800 ms SOA”;

“SOA_800_incon _ nonthreatening” means “mean RT of trails where non-threatening in-group faces’ gaze direction and target location were incongruent at the 800 ms SOA”;

“SOA_800_con _threatening” means “mean RT of trails where threatening out-group faces’ gaze direction and target location were congruent at the 800 ms SOA”;

“SOA_800_incon_threatening” means “mean RT of trails where threatening out-group faces’ gaze direction and target location were incongruent at the 800 ms SOA”.

Table_S3. Data of accuracy in study *a*.

ID	SOA_200_ con _ nonthreatening	SOA_200_ incon _ nonthreatening	SOA_200_ con _ threatening	SOA_200_ incon _ threatening	SOA_800_ con _ nonthreatening	SOA_800_ incon _ nonthreatening	SOA_800- con _ threatening	SOA_800_ incon _ threatening
1	1.0	1.0	1.0	1.0	1.0	1.0	1.0	1.0
2	1.0	1.0	1.0	1.0	1.0	1.0	1.0	1.0
3	1.0	0.97	1.0	1.0	1.0	1.0	0.97	1.0
4	1.0	1.0	1.0	0.97	1.0	1.0	1.0	1.0
5	1.0	0.97	1.0	1.0	1.0	1.0	1.0	1.0
6	0.97	1.0	1.0	1.0	1.0	1.0	1.0	1.0
7	0.84	0.88	0.91	0.94	0.97	0.97	0.94	0.97
8	1.0	1.0	1.0	1.0	1.0	1.0	1.0	1.0
9	1.0	1.0	0.97	1.0	1.0	1.0	1.0	1.0
10	0.97	0.97	0.97	0.91	1.0	0.97	0.97	0.97
11	1.0	0.97	0.94	0.97	0.97	1.0	0.97	1.0
12	0.97	0.94	0.94	0.94	1.0	1.0	1.0	1.0
13	1.0	1.0	0.91	1.0	0.97	1.0	1.0	1.0
14	0.97	0.97	1.0	0.97	1.0	1.0	1.0	0.94
15	1.0	1.0	0.97	1.0	1.0	1.0	1.0	1.0
16	0.97	1.0	0.94	1.0	0.94	1.0	0.94	1.0
17	1.0	1.0	1.0	1.0	1.0	1.0	1.0	1.0
18	1.0	1.0	0.97	0.97	1.0	1.0	1.0	1.0
19	1.0	0.97	1.0	1.0	1.0	1.0	1.0	0.97
20	1.0	1.0	0.94	1.0	1.0	1.0	0.97	1.0
21	1.0	1.0	0.97	1.0	1.0	1.0	1.0	1.0

Note:

"SOA_200_con _ nonthreatening" means "the percentage of correct trails where non-threatening in-group faces' gaze direction and target location were congruent at the 200 ms SOA";

"SOA_200_incon_nonthreatening" means "the percentage of correct trails where non-threatening in-group faces' gaze direction and target location were incongruent at the 200 ms SOA";

"SOA_200_con_threatening" means "the percentage of correct trails where threatening out-group faces' gaze direction and target location were congruent at the 200 ms SOA";

"SOA_200_incon_threatening" means "the percentage of correct trails where threatening out-group faces' gaze direction and target location were incongruent at the 200 ms SOA";

"SOA_800_con _ nonthreatening" means "the percentage of correct trails where non-threatening in-group faces' gaze direction and target location were congruent at the 800 ms SOA";

"SOA_800_incon_nonthreatening" means "the percentage of correct trails where non-threatening in-group faces' gaze direction and target location were incongruent at the 800 ms SOA";

"SOA_800_con _threatening" means "the percentage of correct trails where threatening out-group faces' gaze direction and target location were congruent at the 800 ms SOA";

"SOA_800_incon_threatening" means "the percentage of correct trails where threatening out-group faces' gaze direction and target location were incongruent at the 800 ms SOA".

Table_S4. Data of emotion measure (5-point scale) and the sense of intergroup threat in study *b*.

ID	gender	age	pre_ worried	pre_ anxious	pre_ irritable	pre_ angry	pre_ afraid	post_ worried	post_ anxious	post_ irritable	post_ angry	post_ afraid	score_ threat
1	1	19	1	1	1	1	1	1	1	1	1	1	4
2	1	20	2	1	1	1	1	2	2	1	1	1	2
3	2	22	4	2	1	1	3	2	2	1	1	2	3
4	1	19	1	1	1	1	1	1	1	1	1	1	2
5	1	21	2	1	1	1	1	2	2	1	1	1	2
6	1	20	1	1	1	1	1	1	1	1	1	1	4
7	1	19	1	1	1	1	1	1	1	1	1	1	2
8	1	19	1	1	1	1	1	1	1	1	2	1	3
9	1	19	1	1	1	1	1	2	2	1	1	2	2
10	2	22	3	2	2	3	3	2	3	2	1	2	3
11	1	21	3	3	1	2	2	2	2	1	1	1	2
12	2	27	2	2	1	1	2	2	3	1	2	2	3
13	1	22	1	1	1	1	1	2	1	1	1	1	2
14	2	23	1	1	1	1	1	1	1	1	1	1	2
15	2	25	2	2	1	1	1	1	1	1	1	1	2
16	2	21	1	1	1	1	1	1	2	1	1	1	1
17	1	22	2	2	1	1	2	1	1	1	2	1	2
18	2	22	2	1	2	2	2	1	2	2	2	1	6
19	2	21	1	1	1	1	1	1	1	1	1	1	1

Note:

“ID” means “identification of participants”;

“gender”: “1” means ”female”; “2” means ”male”;

“pre_worried” means “pretest score of worried emotion”

“pre_ anxious” means “pretest score of anxious emotion”

“pre_ angry” means “pretest score of angry emotion”

“pre_ irritable” means “pretest score of irritable emotion”

“pre_ afraid” means “pretest score of afraid emotion”

“post _worried” means “posttest score of worried emotion”

“post _ anxious” means “posttest score of anxious emotion”

“post _ angry” means “posttest score of angry emotion”

“post _ irritable” means “posttest score of irritable emotion”

“post _ afraid” means “posttest score of afraid emotion”

“score_ threat” means “the score of the sense of intergroup threat”.

Table_S5. Mean Reaction time in the gaze-cuing task in study *b*.

ID	SOA_200_ con _in (ms)	SOA_200_ incon _ in (ms)	SOA_200_ con _out (ms)	SOA_200_ incon _out (ms)	SOA_800_ con _in (ms)	SOA_800_ incon _in (ms)	SOA_800- con _out (ms)	SOA_800- incon _out (ms)
1	653.26	675.26	622.23	673.53	653.77	678.86	612.33	617.61
2	781.97	777.59	839.75	740.97	637.78	707.44	686.13	712.16
3	700.84	714.16	701.84	730.74	698.81	692.88	677.06	686.81
4	633.69	632.71	620.63	628.9	606.06	649.26	569.74	616.41
5	759.47	802.44	750.41	760	749.41	766.59	721.56	722.16
6	673.19	708.63	693.06	716.91	683.63	667.84	655.78	652.42
7	634	687.97	656.68	658	652.29	619.09	647.48	646.77
8	625.73	681	618.77	634.66	572.83	512.5	542.83	562.73
9	582	556.1	583.77	567.56	521.83	528.92	547.04	523.11
10	601.22	595.81	577.13	587.28	525.5	569	570.84	560.56
11	721.16	694.06	739.72	727.69	675.25	672.38	643.59	677.75
12	705.09	702.06	703.31	724.61	692.72	729.77	705.56	686.66
13	606.23	616.52	591.09	674	559.03	575.88	587.09	576.19
14	592.58	584.81	616.7	597.16	540.35	537.72	528.87	533.6
15	601.54	602.97	579.97	587.67	550.67	565.23	553.7	562.26
16	643.06	677.28	650.25	657.25	618.61	645.13	609.03	633.75
17	900.79	815.35	785.25	902.2	854.66	867.39	780.71	901.07
18	750.91	783.81	766.59	877.72	750.42	864.13	674.31	783.65
19	749.16	759.9	738.32	757.28	706.59	690.53	684.5	688.91

Note:

“SOA_200_con _ nonthreatening” means “mean RT of trails where non-threatening in-group faces’ gaze direction and target location were congruent at the 200 ms SOA”;

“SOA_200_incon_nonthreatening” means “mean RT of trails where non-threatening in-group faces’ gaze direction and target location were incongruent at the 200 ms SOA”;

“SOA_200_con_threatening” means “mean RT of trails where non-threatening out-group faces’ gaze direction and target location were congruent at the 200 ms SOA”;

“SOA_200_incon _threatening” means “mean RT of trails where non-threatening out-group faces’ gaze direction and target location were incongruent at the 200 ms SOA”;

“SOA_800_con _ nonthreatening” means “mean RT of trails where non-threatening in-group faces’ gaze direction and target location were congruent at the 800 ms SOA”;

“SOA_800_incon _ nonthreatening” means “mean RT of trails where non-threatening in-group faces’ gaze direction and target location were incongruent at the 800 ms SOA”;

“SOA_800_con _threatening” means “mean RT of trails where non-threatening out-group faces’ gaze direction and target location were congruent at the 800 ms SOA”;

“SOA_800_incon_threatening” means “mean RT of trails where non-threatening out-group faces’ gaze direction and target location were incongruent at the 800 ms SOA”.

Table_S6. Data of accuracy in study *b*.

ID	SOA_200_ con _in	SOA_200_ incon _ in	SOA_200_ con _out	SOA_200_ incon _out	SOA_800_ con _in	SOA_800_ incon _in	SOA_800- con _out	SOA_800- incon _out
1	0.97	0.97	0.97	0.94	0.94	0.88	0.94	0.97
2	1	1	1	1	1	1	1	1
3	0.97	1	1	0.97	1	1	1	1
4	1	0.97	0.94	0.94	1	0.97	0.97	1
5	1	1	1	1	1	1	1	1
6	1	1	1	1	1	1	1	0.97
7	0.97	0.97	0.97	0.97	0.97	1	0.97	0.94
8	0.81	0.94	0.97	0.91	0.94	1	0.94	0.94
9	0.91	0.91	0.81	0.78	0.75	0.78	0.81	0.84
10	1	1	1	1	1	1	1	1
11	1	1	1	1	1	1	1	1
12	1	1	1	0.97	1	0.97	1	1
13	0.97	0.97	1	0.97	1	1	1	0.97
14	0.97	0.97	0.94	0.97	0.97	0.91	0.97	0.94
15	0.88	0.91	0.94	0.94	0.94	0.94	0.84	0.97
16	1	1	1	1	0.97	1	1	1
17	0.91	0.97	0.88	0.94	0.91	0.88	0.88	0.94
18	1	1	1	1	0.97	1	1	0.97
19	1	0.97	0.97	1	1	1	1	1

Note:

“SOA_200_con_in” means “the percentage of correct trails where non-threatening in-group faces’ gaze direction and target location were congruent at the 200 ms SOA”;

“SOA_200_incon_in” means “the percentage of correct trails where non-threatening in-group faces’ gaze direction and target location were incongruent at the 200 ms SOA”;

“SOA_200_con_out” means “the percentage of correct trails where non-threatening out-group faces’

gaze direction and target location were congruent at the 200 ms SOA”;

“SOA_200_incon_out” means “the percentage of correct trails where non-threatening out-group faces’ gaze direction and target location were incongruent at the 200 ms SOA”;

“SOA_800_con_in” means “the percentage of correct trails where non-threatening in-group faces’ gaze direction and target location were congruent at the 800 ms SOA”;

“SOA_800_incon_in” means “the percentage of correct trails where non-threatening in-group faces’ gaze direction and target location were incongruent at the 800 ms SOA”;

“SOA_800_con_out” means “the percentage of correct trails where non-threatening out-group faces’ gaze direction and target location were congruent at the 800 ms SOA”;

“SOA_800_incon_ou” means “the percentage of correct trails where non-threatening out-group faces’ gaze direction and target location were incongruent at the 800 ms SOA”.
